# Potential Phytotoxic Effect of Essential Oil of Non-Native Species *Impatiens parviflora* DC.

**DOI:** 10.3390/plants8070241

**Published:** 2019-07-23

**Authors:** Jana Jurová, Martina Matoušková, Anna Wajs-Bonikowska, Danuta Kalemba, Marek Renčo, Vincent Sedlák, Zuzana Gogaľová, Janka Poráčová, Peter Šalamún, Daniela Gruľová

**Affiliations:** 1 Institute of Parasitology, Slovak Academy of Sciences, Hlinkova 3, 04001 Košice, Slovakia; 2 Institute of General Food Chemistry, Lodz University of Technology, Stefanowskiego 4/10 St., 90924 Łódz, Poland; 3 Department of Biology, Faculty of Humanities and Natural Sciences, University of Prešov, 17. Novembra 1, 08001 Prešov, Slovakia

**Keywords:** allelopathy, biological activity, biocides, *Impatiens parviflora*, small balsam, essential oil, extract

## Abstract

*Impatiens parviflora* is non-native invasive plant species occupying large areas all over the Europe and threatens native communities by altering their species composition and reducing native biodiversity. The factor responsible for its spreading could be explained by releasing biochemical to the environment. On the other hands, high demand on secondary metabolites as potential source of new ecofriendly biocides could be beneficial. The analysis of *I. parviflora* essential oil (EO) led us to identify more than 60 volatiles. The main compound was hexahydrofarnesyl acetone, other dominant components were phytol, carvacrol, germacra-4(15),5,10(14)-trien-1-α-ol, and pentacosane. The potential phytotoxic effect of *I. parviflora* EO collected in two vegetation periods (summer and autumn) was evaluated on seed germination and root elongation of three dicot species (*Raphanus sativus*, *Lepidum sativum*, and *Lactuca sativa*) and on one monocot species (*Triticum aestivum*). The seed germination of only one dicot species, *L. sativa*, was affected by both EOs. In contrast, seed germination of monocot species *T. aestivum* was influenced only by the highest doses of EOs isolated from *I. parviflora* in autumn. The root elongation of tested plant species was less influenced by *I. parviflora* EOs. *L. sativum* showed sensitivity to one dose of EOs hydrodistilled in summer, while the monocot species was influenced by both EOs samples in highest doses. Our findings revealed that *I. parviflora* contained phenolics that were phytotoxic to the germination of some plant species, mainly at higher EOs doses, while root elongation of tested plants was not suppressed by essential oils.

## 1. Introduction

*Impatiens parviflora* DC. (small balsam) belongs to the Balsaminaceae family and is native to Eastern Siberia. The species spread in many countries cross the Europe as well as in Canada [1,2,3]. Slovak State Nature Protection agency and the European Union categorize this species as a non-native species. Different studies focus on its distribution and ecology as invasive species [3,4,5,6,7,8]. *I. parviflora* undoubtedly has an environmental influence on native biodiversity through changes in community structure, nutrient cycles, trophic levels, hydrology, and competition act as an invasive species [6,9]. According to Slovak legislation, *I. parviflora* is considered as a dangerous species with the potential to adversely affect natural ecosystems. It is assumed that it was introduced to the Europe by trading as ornamental plant as most of the invasive species. First observation of the *I. parviflora* in Europe is dated to 1890 [1]. In the beginning it occurred in botanical gardens, parks and cemeteries. Escape to the wild started in Germany in early 19th century [10,11].

*I. parviflora* is an annual herb. The stem is typically bare and straight with a tendency to widening at the nodes [1]. The germination usually ends until the beginning of May. The growth in length is most intense from the middle of May to middle of June and grows to a height on average from 30 to 80 cm. Leaves are typically alternate and ovate. Flowers are zygomorphic, 7–15 mm long and white-yellow colorued with occasional thin red patterns [12]. Individual plants under favourable conditions can produce up to ten thousand seeds in the three months of the fruit growing. The main fruit ripening period of the *I. parviflora* is in August [1]. Seeds have ballistic form of dispersal and after the slight touch to the ripened fruits or in stronger wind they are spread to the surrounding [13].

*I. parviflora* grows on various mineral soils from the strongly acidic through the acidic to the slightly calcareous soils with pH range of 4.5 to 7.6 [14]. Plants prefer wet, shaded, or half-shaded places within a wide range of habitats such as parks, gardens, along roads, on riversides, railway embankments, and cauliflower fields, as well as in various forests, forest clearings, paths, and edges, and disrupts natural plant communities in many locations [8,15,16].

There are many theories why invasive plants have success and spread easy in new environment. One of the important factors is considered by the production and releasing of allopathic compounds [17]. Secondary metabolites of introduced plant species have been identified as phytotoxic for surrounded plant population [18]. This fact is known as allelopathy and numerous studies suggest that it plays important role in succession of plant invasion [19]. Identification of chemical compounds produced by alien species can serve better understanding of their impact on the local environment. Besides this, interest about invasive species increases in level of recognition their chemical components which could present potential source of green pesticides [20]. Till now allelopathy effect of different extracts from *I. parviflora* based on model plant species (*Brassica napus, Leucosinapis alba* and *Triticum aestivum*) were investigated. The chemical composition of *I. parviflora* water or ethanol extracts have been studied in several previous studies, but chemical composition of essential oil of *I. parviflora* (EO) is known only from one population in Poland (Table 1). To our best of knowledge however, potential allelopathy of EO from *I. parviflora* was not yet studied.

The aim of the present work was to determine (1) the content and composition of EO of *I. parviflora* isolated from the population collected in Slovakia twice within vegetation season and (2) the effect of different EO doses on the seeds germination and root elongation of four model plant species (*Raphanus sativus* L., *Lepidium sativum* L., *Lactuca sativa* L., and *Triticum aestivum* L.). We hypothesized variability in chemical composition of EO hydrodistilled from Slovak and Poland population of *I. parviflora* due to different climatic conditions, extraction methods, growth stage, etc., as well as different impact of EO doses on seed germination and root elongation of some test plant species.

## 2. Results

### 2.1. Content and Composition of EO

Total amount of EO hydrodistilled from the dried samples of *I. parviflora* collected in June (IP1) and in September (IP2) presented 0.11% and 0.14% of dry mass, respectively. In total, 61 compounds in sample IP1 and 64 compounds in IP2 were identified, which presented 86.9% and 71.5% of total components (Table 2), respectively. Chemical composition of EOs isolated from *I. parviflora* were, however, considerably diverse between sampling season. If we consider the composition of examined essential oils they are really very diverse. Both essential oils were dominated by numerous as well of high content group of oxygenated derivatives of terpene and non-terpene structure volatiles. Among them several highly biologically active molecules like monoterpene phenol and alcohols of monoterpene structure, e.g., carvacrol and linalool; sesquiterpene and diterpene structure (nerolidol, phytol, and isophytol), aliphatic non-terpene alcohols, e.g., oct-1-en-3-ol, nonan-1-ol etc.; oxides (2-pentylfuran, two isomers of rose oxide, β-ionone epoxide); and oxides of the sesquiterpene skeleton such as caryophyllene and ledane skeleton were revealed. Detected volatiles composed also of numerous short or long chain aliphatic aldehydes, e.g., hexanal, (*E,E*)-deca-2,4-dienal), and aliphatic or sesquiterpene ketones (5-ethyl-6-methylhept-3*E*-en-2-one or salvial-4(14)-en-1-one). Surprisingly, we were able to identify in EOs besides of rose oxides the C(13)-norisoprenoids like (E)-β-damascenone and α- and β-ionone and its derivatives—volatiles—which are mainly characteristic for valuable aroma plants. As a main compound in both samples, hexahydrofarnesyl acetone was identified, the amount of which doubled within the vegetation season (15.7% in IP1 and 31.0% in IP2). Other dominant components identified in sample IP1 were phytol (12.3%), carvacrol (10.9%), and germacra-4(15),5,10(14)-trien-1-α-ol (9.4%), followed by pentacosane (4.9%). Their amounts however decreased in autumn to 5.2% (phytol), 0.4% (carvacrol), or 0.5% (germacra-4(15),5,10(14)-trien-1- α-ol). Both EOs were also characterized by high content of long-chain alkanes–molecules characteristic for plant material of very low essential oils content, among 16 identified hydrocarbons the dominant one was pentacosane, its amount in IP1 and IP2 samples was stabile (5.2%) (Table 2).

### 2.2. Phytotoxic Effect

The biological effect of EO hydrodistilled from *I. parviflora* was investigated in different months (June and September) from the same locality. There were effects on (a) seed germination and (b) root elongation of three dicot species (*Raphanus sativus* L., *Lactuca sativa* L., and *Lepidium sativum* L.) and one monocot species (*Triticum aestivum* L.). In general, no significant effect of all applied EOs concentrations of *I. parviflora* was found on seed germination of *R. sativus* and *L. sativum* (*p* = 0.05). In contrast, seed germination of *L. sativa* was significantly reduced by different concentrations of EOs in comparison to the untreated control. Significant effect of inhibition of seed germination was evident in all concentrations of EOs extracted from *I. parviflora* collected in June (IP1) (*p* = 0.05). The highest impact was found at the 0.125 μg/mL EO concentration, where the number of germinated seeds was ~50% lower (5.0 ± 1.0 germinated seeds) in comparison to control. However, treatments with IP2 EOs significantly reduced *L. sativa* seed germination only at doses 0.125, 0.250, and 1.250 μg/mL (*p* = 0.05).

Inhibition of seed germination of monocot plant *T. aestivum* was also found. Significant reduction was recorded in two highest concentrations of IP2 EO (1.250 and 2.50 μg/mL) (*p* = 0.05) (Table 3).

Root elongation of *L. sativum* was significantly reduced, but only at one applied dose (0.625 μg/mL) of IP1 EO. In contrast to negative impact of tested doses of EO on seed germination, no inhibitory effect on root elongation of *L. sativa* and *R. sativus* has been recorded (*p* = 0.05). The highest applied dose (2.5 μg/mL) of IP1 an IP2 EOs significantly inhibited root elongation of monocot plant seeds of *T. aestivum* (*p* = 0.05) (Table 4). Besides the expected inhibitory effect of tested EOs on root elongation of tested plants, a moderate stimulatory effect of some EO concentrations was found, however without statistical differences.

There have not been found significant influence of the seasonal changes in EO composition and germination or root elongation of model plants. Different EOs and different concentrations have variable influence on different plant species.

## 3. Discussion

To the best of our knowledge, this is the first study that has investigated the biological activity of EO and the second study to analyze the composition of the EO of *I. parviflora*. Our results can be useful to explain the ecological role of *I. parviflora*, and its EO could be considered as a potential factor contributing to its spread in new areas. Surprisingly, the content and composition of *I. parviflora* EO collected in Slovakia differs from Poland [28]. While the yield of EO (w/w relative to dry material weight) of *I. parviflora* from Poland was 0.24% [28], the amount of *I. parviflora* EO collected in Slovakia was much lower (0.11–0.14%). Species of the genus *Impatiens* are not essential oil bearing plant; they contain only traces of volatiles. Dominant compounds identified in Polish population of *I. parviflora* EO were (E)-hex-3-en-1-ol (16.8%), linalool (15.1%), benzaldehyde (10.2%), followed by hexanal (5.3%) and hexanol (4.9%); while in Slovak population only linalool (1.1% in IP1 and 0.2% in IP2) and hexanal (0.2% in sample IP2) were found, but in very small concentration. Interestingly, we revealed presence of safranal in *I. parviflora* essential oils, allelochemical mainly present in saffron plants [31].

Based on this and previous study [28] we can observe strong differences in essential oil composition not only between selected *Impatiens* species, but also within one species, growing in a different areas (Slovakia and Poland [28]). If we compare the volatiles detected in current and already published study [28], we can observe that the *I. parviflora* oils are composed mainly of oxygenated compounds; in the examined oils, mainly oxygenated derivatives of sesquiterpene and monoterpene hydrocarbons as well of C13-norisoprenides, while in a previous study of oxygenated compounds, aliphatic hydrocarbons (42.8%) and monoterpenes (24.7%). What is interesting is that some C13-norisoprenides, like β-ionone and it epoxide as well *cis*- and *trans*-rose oxides, were identified in both oils. Moreover, hexahydrofarnesyl acetone—the main volatile in EO of current study (15.7% and 31.0%)—was also observed in oil isolated from plant growing in Poland, but in much lower quantity (0.4%). From the main compounds—hex-3*E-*en-1-ol (16.8%), linalool (15.1%), benzaldehyde (10.2%), hexanal (5.3%) and hexanol (4.9%) identified in a previous study [28]—only linalool and hexanal were detected in the examined essential oils. A common feature of *I. parviflora* oils is that numerous groups of *n*-alkanes, as well of non-terpene aliphatic alcohols, aldehydes, esters, and acids, were detected.

The dominant compound, hexahydrofarnesyl acetone, identified in our samples have had only 0.4% content in Poland population of *I. parvilfora* [28], but was dominant in EO hydrodistillated in *Hippuris vulgaris*, which is in high risk of extinction in Italy [32], as well as in medicinal species *Impatiens balsamina* [28]. As main components were identified also in other plant species, for example in the SDE extract from the flowers of *Trifolium repens* L. grown in Poland [33], in fresh needles and branches of *Torus baccata* L. in Serbia (Gymnospermae) [34], or in leaves and flowers of *Zizyphus jujube* from Egypt [35].

The composition of EO highly depends on climatic conditions, plant growth stage, collection time, drying conditions, distillation method, etc. [36]. Within two collections (IP1 and IP2) in the 2017 vegetation season there have been observed changes in quantity and quality of EO. Considerable differences in the qualitative and quantitative composition of the EOs obtained from the same plant species of different ages were also revealed in several previous studies [37,38,39]. However, the total character of EO is influenced by genetic factors confirming that the potential to produce a certain chemical pattern is genetically coded, but the gene expression will be introduced or also repressed by environmental factors [40,41].

The allelopathic effect of *I. parviflora* water extracts of the aerial plant parts was already observed in *Sinapis alba* seeds [1,30]. Similar, the water and methanol extracts from familiar species *I. glandulifera* and *I. noli-tangere* on seed germination of *Brassica napus* as well as *S. alba* was evaluated [30]. The effect of the water extract of *I. glandulifera* was also compared on germination and seedling growth in *B. napus*, dicot, and *Triticum aestivum*, monocot [17]. Inhibition by those extracts was recorded stronger for *B. napus* than that for *T. aestivum.* Previous findings are in accordance with our data when seed dicot species *L. sativa* in germination and *R. sativus* in root elongation were more sensitive than monocot *T. aestivum*. A possible reason for this difference is various seed coat anatomies and thus its permeability.

The phytotoxic effect of the EO of different plant species is generally attributed to the presence of the main components. As there is no evidence about the hexahydrofarnesyl acetone phytotoxicity, the results of several previous studies show the significant influence of carvacrol and linalool. Herbicidal properties of *Zataria multiflora* EO are attributed to their major components carvacrol and linalool [42] as well as carvacrol along with thymol tested as a standard compounds were suggested as alternative pesticides, herbicides, as well as insecticides [43]. Comparable studies were done to evaluate phytotoxic effect of EO of invasive or non-native plants. Five populations of invasive goldenrod species (*Solidag gigantea* and *Solidago canadensis*) were investigated in east Slovakia. The samples significantly inhibited seeds germination of *L. sativa* while adverse effect was observed when *Solidago* spp. EO significantly stimulated the radical elongation of *R. sativus* as well as *L. sativa* [44]. Another study evaluated phytotoxicity of volatiles produced by alien species *H. mantegazzianum* to model plants. EO hydrodistilled from the alien plant species presented biological activity on the dicot and monocot plant species. The most sensitive was *Lactuca sativa* compared to *Lepidium sativum* and *Raphanus sativus* in seed germination as well as in root length elongation. Stimulation effect was visible in both root length and root number at two or one highest doses, respectively in monocot species *Triticum aestivum* L. [45].

Besides the experiments which could collaborate to explain important ecological role of invasive or non-native plant species, there is also interest to study their pesticide effect as a great source for ecopesticides/ecoherbicides. In the same way, *I. parviflora* evaporated methanolic extract was tested against green peach aphid (*Myzus perssicae*), an important insect pest of many plants [46]. *I. parviflora* repelled the aphid strongly and significantly. Other examples are *Solidago gigantea* and *Solidago canadensis*, two invasive weeds, which EOs have been also exploited as a potential source of green pesticides against *Culex quin quefasciatus*, *Spodoptera littoralis* and *Musca domestica.* Deep research of natural–cides, which are now known to be effective, selective, biodegradable, and less toxic to environment, would help decrease the negative impact of synthetic agents in environmental pollution [43]. EOs had been recognized as prospective, environmentally acceptable and active ingredients employed in IPM [20].

## 4. Materials and Methods 

### 4.1. Plant Material

Non-native species *Impatiens parviflora* DC. was collected from a productive, undisturbed deciduous forest ~100 years old, near the village of Skároš in south eastern Slovakia, central Europe (48°36.16’N, 21°23.37’E; 370 m a.s.l.). Plant material was identified by Marek Renčo (Institute of Parasitology, Slovak Academy of Sciences). Voucher samples have been stored in the herbarium of Department of Ecology of University of Presov, number IP1 and IP2_224/2017. The tree canopy mainly consisted of *Fagus sylvatica* (65%), *Quercus robur* (25%), and *Acer pseudoplantanus* (10%), without shrub species. Aboveground vegetation dominated by *Impatiens parviflora* grown in soil with pH 3.4 to 5.2. This region has a temperate climate, with an annual average of 40 summer days and a warm, moderately dry subregion with a mild winter. The mean annual precipitation is 650–700 mm. The soils are characterized as Cambisols.

Collection was done two times within the vegetation season 2017 in different vegetation period. First collection was done on June 1st (IP1) when the first flowers generally show up. Second collection was done on September 17th (IP2) when the flowering and ripening seeds finalize. Plant material was dried in laboratory condition on thin layer on filtrate paper for 2 weeks until crushing. Thereafter it was placed for EO isolation.

### 4.2. EO Isolation

Approximately 30 g of dried aerial plant parts of each sample were cut in small pieces and subjected to hydrodistillation for 3 h in a Clevenger-type apparatus. Essential oils were stored in dark vials until tested and analyzed.

### 4.3. GC-MS Analysis

Essential oils were analyzed by GC-MS-FID in Institute of General Food Chemistry, Lodz University of Technology, Poland). The analysis was performed on a Trace GC Ultra coupled with DSQII mass spectrometer (Thermo Electron, Waltham, MA, USA). A simultaneous GC-FID and MS analysis was performed using a MS-FID splitter (SGE Analytical Science, Ringwood Victoria, Australia). Mass range was 33 to 550 amu; ion source heating: 200 °C; ionization energy: 70 eV. One microliter of essential oil solution (80% *v/v*) diluted in pentane: diethyl ether was injected in split mode at split ratios (50:1). Operating conditions for capillary column Rtx-1 MS (60 m × 0.25 mm i.d., film thickness 0.25 µm), and temperature program: 50 (3 min)–300 °C (30 min) at 4 °C/min. Injector and detector temperatures were 280 °C and 300 °C, respectively. Carrier gas was helium (constant pressure: 300 kPa).

### 4.4. Compounds Identification 

The identification of compounds was based on a comparison of their mass spectra (MS) and linear retention indices (RIs, nonpolar column), determined with reference to a series of alkanes C8–C26, by comparing with those in MassFinder 3 [47,48] as well as with computer mass libraries NIST 2012 and the Wiley Registry of Mass Spectral Data 8th edition.

### 4.5. Model Plants

Potential phytotoxic effect of *I. parviflora* EO was evaluated on seeds of four species. Three dicotyledonous species—*Raphanus sativus* L. (radish), *Lepidium sativum* L. (garden cress), and *Lactuca sativa* L. (lettuce)—and one monocotyledonous species, *Triticum aestivum* L. (common wheat), were chosen as model plants. *R. sativus* var. radicula Pers. (cv. ’Duo’), *L. sativum* (cv. ’Dánska’), and *L. sativa* (cv. Král Máje I.) seeds were purchased from Zel Seed (Slovakia). Common wheat was obtained from the Research Center in Malý Šariš.

### 4.6. Phytotoxic Activity Assay

Phytotoxic assay followed previously used method by Mancini et al. (2014) [49]. Three factors were taken into account in the experimental treatment: (i) four test plants [radish (*R. sativus* L.), garden cress (*L. sativum* L.), lettuce (*L. sativa* L.), and common wheat (*T. aestivum* L.)]; (ii) *I. parviflora* EOs from two periods of vegetation season; and (iii) six different concentrations of *I. parviflora* EO [ 2.5, 1.25, 0.625, 0.25, 0.125, and 0.062 g/mL]. EOs were dissolved in distilled water/acetone 99.5:0.5 and diluted to prepare the desired concentrations. Distilled water/acetone 99.5:0.5 was used as control. Test seeds were surface sterilized in 95% EtOH for 15 s and rinsed triplicate in distilled water. Ten sterilized seeds were sown into each Petri dish (90 mm diameter) containing 5 layers of Whattman filter paper. In each Petri dish 7 mL of EO solution of different concentration or distilled water/acetone 99.5:0.5 was added. Each treatment was triplicated. The Petri dishes were kept in growth chamber (20 ± 1 °C, natural photoperiod, Sanyo, MLR-351H). Evaluation of germination and the radicle length (cm) was measured after 120 h.

### 4.7. Statistical Analysis

Data from the experiment were subjected to analysis of variance (ANOVA) by Least Significant Difference Test. Statistical analyses were performed using the PlotIT ver. 3.2 program (Scientific Programming Enterpises, Haslett, MI, USA).

## 5. Conclusions

*I. parviflora* is one of the most widespread invasive plants in the temperate and northern regions of Europe, especially in forest habitats. Our results showed that content and chemical composition of EOs isolated from tissues of *I. parviflora* (Slovak population) varied within vegetation season and was considerably different from those revealed in population from Poland. The EOs treatments at different doses affected seed germination of selected plant species, while root elongation was considerable less affected. The presence of carvacrol, one of the main compounds identified in EOs, whose phytotoxic effect was already manifested, shows that EO can be attributed as a possible factor responsible for the easy spreading of this invasive plant species. On the other hand, invasive plant species could present great source of the chemicals potentially used as ecofriendly biopesticides (herbicides, insecticides, fungicides, and bactericides), e.g., hexahydrofarnesyl acetone found as dominant compound in our *I. parviflora* plants. Future studies with hexahydrofarnesyl acetone may hold the answer to this question.

## Figures and Tables

**Table 1:** Overview table with the up-to-date publications focusing on chemical composition of *Impatiens parviflora* extract.

References	Country	Solvents	Chem. Groups	Allelopathy	Tested Plant Model
[17]	Lithuania	H_2_O	phenols	yes	*Brassica napus* *,*
					*Triticum aestivum*
[21,22,23]	Poland	HCl_3_, MeOH	triterpenoid saponins,	no	
			galactolipids		
[24]	UK	EtOH	acid-soluble proteins	no	
[25,26,27]	Poland	EtOH, H_2_O, HCl_3_, MeOH, C_3_H_6_O	water-soluble	no	
			phenolic acids,		
			phenolic acids,		
			polysacharides,		
			polyphenols		
[28]	Poland	H_2_O	essential oil - diverse	no	
			chemical groups		
[29]	Czech Rep.	HCl_3_, MeOH, EtOh	polysaccharides		
	Slovak Rep.				
[30]	Czech Rep.	MeOH, CH_2_Cl_2_		yes	*Leucosinapis alba,*
					*Brassica napus*

**Table 2:** Identified components of two samples of *Impatiens parviflora*.

		RI^exp.^	RI^lit^.	IP1	IP2
No.	Compounds			[%]	
1	**hexanal**	773	776	n.i.	0.2
2	**octane**	797	800	n.i.	0.1
3	**1,6-dimethylhepta-1,3,5-triene**	833	837	0.2	tr
4	**heptanal**	876	879	n.i.	0.4
5	**nonane**	898	900	n.i.	0.2
6	**oct-1-en-3-ol**	963	963	n.i.	0.2
7	**2-pentylfuran**	977	981	0.1	0.5
8	**octanal**	979	981	0.4	0.3
9	**p-cymene**	1012	1015	2.9	2.2
10	**limonene**	1021	1025	0.1	0.1
11	**(*E*)-β-ocimene**	1045	1041	0.7	0.3
12	**terpinolene**	1075	1082	3.0	1.1
13	**nonanal**	1080	1084	0.4	1.9
14	**linalool**	1081	1086	1.1	0.2
15	***cis*-rose oxide**	1093	1110	0.2	n.i.
16	***trans*-rose oxide**	1109	1124	0.1	0.1
17	**(*E*)-5-ethyl-6-methylhept-3-en-2-one**	1122	1124	n.i.	0.3
18	**nonan-1-ol**	1155	1152	0.2	0.2
19	***trans*-p-mentha-1(7),8-dien-2-ol**	1154	1155	n.i.	0.2
20	**p-cymen-9-ol**	1158	1157	0.2	0.5
21	**octanoic acid**	1165	1164	0.1	n.i.
22	**safranal**	1169	1173	0.4	tr
23	**hexyl butyrate**	1170	1173	n.i.	1.1
24	**decanal**	1180	1180	0.5	0.8
25	**(*E*)-dec-2-enal**	1239	1236	0.2	0.4
26	**nonanoic acid**	1259	1260	0.2	n.i.
27	**carvacrol**	1283	1278	10.9	0.4
28	**(*E,E*)-deca-2,4-dienal**	1289	1288	n.i.	0.2
29	**(*E*)-undec-2-enal**	1337	1338	tr	0.8
30	**decanoic acid**	1355	1350	0.2	n.i.
31	**(*E*)-β-damascenone**	1358	1363	0.1	0.1
32	**(*E*)-undec-2-en-1-ol**	1375	1368	0.1	0.2
33	**(*E*)-β-bourbonene**	1379	1386	0.3	0.1
34	**hexahydropseudoionone**	1384	1391	0.3	1.0
35	**tetradecane**	1395	1400	n.i.	0.3
36	**α-ionone**	1402	1409	0.4	0.3
37	**(E)-β-caryophyllene**	1413	1420	0.5	n.i.
38	**geranylacetone**	1426	1430	0.2	0.2
39	**α-humulene**	1446	1455	0.1	0.1
40	**dehydro-β-ionone**	1453	1460	0.5	0.5
41	**β-ionone epoxide**	1456	1460	0.9	0.8
42	**β-ionone**	1459	1468	1.3	2.1
43	**germacrene D**	1471	1479	2.5	1.0
44	**tridecanal**	1486	1486	0.4	0.3
45	**germacrene A**	1493	1503	n.i.	0.4
46	**γ-cadinene**	1501	1507	0.1	0.3
47	**calamenene**	1506	1517	0.2	n.i.
48	**δ-cadinene**	1509	1520	0.6	0.1
49	**(E)-nerolidol**	1543	1553	0.1	0.1
50	**3,7,11-trimethyldodecan-1-ol**	1549	1563	0.4	0.6
51	**caryophyllene oxide**	1565	1578	1.3	0.3
52	**salvial-4(14)-en-1-one**	1573	1592	0.5	0.2
53	**tetradecanal**	1587	1586	n.i.	0.3
54	**widdrol**	1620	1618	0.3	0.4
55	**ledene oxide (II)**	1657	1646	0.3	0.3
56	**germacra-4(15),5,10(14)-trien-1-α-ol**	1665	1680	9.4	0.5
57	**benzyl benzoate**	1720	1720	2.1	0.4
58	**hexahydrofarnesyl acetone**	1826	1833	15.7	31.0
59	**hexadecanoic acid**	1956	1958	n.i.	3.8
60	**nonacosane**	1899	1900	0.3	n.i.
61	**isophytol**	1931	1938	0.7	n.i.
62	**ethyl hexadecanoate**	1977	1992	0.3	n.i.
63	**phytol**	2095	2114	12.3	5.2
64	**docosane**	2197	2200	n.i.	0.2
65	**tricosane**	2289	2300	1.5	1.9
66	**tetracosane**	2388	2400	0.5	0.5
67	**pentacosane**	2495	2500	4.9	5.2
68	**hexacosane**	2593	2600	0.6	n.i.
69	heptacosane*		2700	1.1	2.1
70	octacosane*		2800	0.7	n.i.
71	nonacosane*		2900	0.9	1.3
72	triacontane*		3000	0.6	0.4
73	hentriacontane*		3100	1.3	0.7
74	dotriacontane*		3200	0.4	0.3
75	tritriacontane*		3300	0.3	0.2
	**Monoterpene hydrocarbons**			6.8	5.3
	**Oxygenated derivatives of monoterpene hydrocarbons**			12.9	1.4
	**Sesquiterpene hydrocarbons**			4.4	2.0
	**Oxygenated derivatives of sesquiterpene hydrocarbons**			27.9	33.2
	**Diterpene hydrocarbons**			3.5	4.9
	**Oxygenated derivatives of diterpene hydrocarbons**			n.i.	n.i.
	**C13-norisoprenoides**			13.0	5.2
	**Non-terpene alcohols**			0.8	1.2
	**Non-terpene aldehydes**			2.2	5.6
	**Non-terpene ketones**			tr	0.3
	**Non-terpene esters**			2.4	0.4
	**Non-terpene oxides**			0.1	0.5
	***n*-Alkanes**			12.9	13.2
	***n*-Alkenes**			0.2	tr
	**Carboxylic acid**			0.3	3.8
	**Total identified**			**87.1**	**76.2**

n.i.—not identified; tr < 0.05%, RI ex—experimental retention index on unpolar column; RI lit—literature retention index on unpolar column (Mass Finder 3.0 and NIST 2012 library); *—alkanes identified based on their characteristic mass spectra.

**Table 3:** Effect of different doses of EO from *I. parviflora* on the seed germination of three dicot and one monocot model plant species.

		EO Doses [µg/mL]						
Treated Seeds	Collection Period	Number of Germinated Seeds ± SD						
		0.065	0.125	0.250	0.625	1.250	2.500	Control
*R. sativus*	IP1	9.3 ± 0.6 ^a^	8.7 ± 0.7 ^a^	9.3 ± 0.6 ^a^	9.7 ± 0.5 ^a^	10.0 ± 0.0 ^a^	9.3 ± 0.6 ^a^	9.0 ± 0.8 ^a^
	IP2	10.0 ± 0.0 ^a^	10.0 ± 0.0 ^a^	9.6 ± 0.6 ^a^	9.0 ± 0.5 ^a^	10.0 ± 0.0 ^a^	10.0 ± 0.0 ^a^	10.0 ± 0.0 ^a^
*L. sativa*	IP1	6.6 ± 0.6 ^b^	5.0 ± 1.0 ^b^	6.3 ± 1.5 ^b^	7.3 ± 0.6 ^b^	7.3 ± 0.6 ^b^	7.3 ± 1.2 ^b^	10.0 ± 0.0 ^a^
	IP2	9.0 ± 0.6 ^a^	7.0 ± 0.9 ^b^	7.0 ± 1.1 ^b^	8.0 ± 1.5 ^a^	7.0 ± 1.7 ^b^	8.0 ± 2.0 ^a^	10.0 ± 0.0 ^a^
*L. sativum*	IP1	10.0 ± 0.0 ^a^	9.0 ± 0.7 ^a^	9.7 ± 0.6 ^a^	10.0 ± 0.0 ^a^	10.0 ± 0.0 ^a^	9.3 ± 0.5 ^a^	9.3 ± 0.5 ^a^
	IP2	9.6 ± 0.6 ^a^	10.0 ± 0.0 ^a^	9.6 ± 0.6 ^a^	10.0 ± 0.0 ^a^	10.0 ± 0.0 ^a^	8.0 ± 1.0 ^a^	10.0 ± 0.0 ^a^
*T. aestivum*	IP1	7.3 ± 1.5 ^a^	7.7 ± 1.2 ^a^	6.7 ± 0.6 ^a^	8.3 ± 0.6 ^a^	7.0 ± 1.0 ^a^	7.7 ± 1.2 ^a^	9.3 ± 0.6 ^a^
	IP2	8.3 ± 0.6 ^a^	9.3 ± 0.6 ^a^	9.0 ± 0.0 ^a^	8.0 ± 1.0 ^a^	7.3 ± 0.7 ^b^	5.3 ± 2.2 ^b^	9.0 ± 1.0 ^a^

Each value is an average of 3 replications; SD = standard deviation; the letters a,b,c in each column present statistical differences according to Least Significant Difference Test (*p* = 0.05).

**Table 4:** Effect of different doses of EO of *I. parviflora* on the root elongation of three dicot and one monocot model plant species.

		EO Doses [µg/mL]						
Treated Seeds	Collection Period	Radical Elongation ± SD						
		0.065	0.125	0.250	0.625	1.250	2.500	Control
*R. sativus*	IP1	3.3 ± 1.3 ^a^	2.4 ± 1.2 ^a^	3.1 ± 1. ^a^	2.7 ± 1.0 ^a^	3.0 ± 1.3 ^a^	2.6 ± 1.3 ^a^	3.3 ± 1.4 ^a^
	IP2	3.8 ± 1.3 ^a^	3.8 ± 1.7 ^a^	3.7 ± 1.7 ^a^	2.9 ± 1.3 ^a^	3.2 ± 1.0 ^a^	3.5 ± 1.2 ^a^	3.7 ± 1.4 ^a^
*L. sativa*	IP1	1.2 ± 0.8 ^a^	1.2 ± 0.6 ^a^	1.1 ± 0.6 ^a^	1.4 ± 0.7 ^a^	1.3 ± 0.8 ^a^	1.4 ± 0.7 ^a^	0.9 ± 0.4 ^a^
	IP2	0.8 ± 0.5 ^a^	1.3 ± 0.7 ^a^	0.9 ± 0.6 ^a^	1.1 ± 0.6 ^a^	1.0 ± 0.6 ^a^	0.9 ± 0.5 ^a^	1.0 ± 0.7 ^a^
*L.sativum*	IP1	5.5 ± 2.4 ^a^	6.5 ± 2.6 ^a^	5.9 ± 3.0 ^a^	3.6 ± 1.2 ^b^	6.5 ± 1.9 ^a^	7.6 ± 2.5 ^a^	5.3 ± 2.6 ^a^
	IP2	6.4 ± 2.1 ^a^	6.7 ± 1.7 ^a^	6.3 ± 2.6 ^a^	7.6 ± 1.6 ^a^	6.8 ± 2.4 ^a^	8.1 ± 1.6 ^a^	8.1 ± 1.6 ^a^
*T. aestivum*	IP1	2.4 ± 0.9 ^a^	3.0 ± 0.9 ^a^	2.0 ± 0.9 ^a^	2.4 ± 1.0 ^a^	2.4 ± 1.0 ^a^	1.9 ± 0.5 ^b^	2.8 ± 1.1 ^a^
	IP2	2.1 ± 1.2 ^a^	3.5 ± 1.7 ^a^	2.7 ± 1.0 ^a^	2.8 ± 1.1 ^a^	2.7 ± 1.2 ^a^	1.9 ± 0.8 ^b^	2.8 ± 0.9 ^a^

Each value is an average of 3 replications; SD = standard deviation; the letters a,b,c in each column present statistical differences according to Least Significant Difference’s Test (*p* = 0.05).
